# Identification of Candidate Children for Maturity-Onset Diabetes of the Young Type 2 (MODY2) Gene Testing: A Seven-Item Clinical Flowchart (7-iF)

**DOI:** 10.1371/journal.pone.0079933

**Published:** 2013-11-11

**Authors:** Michele Pinelli, Fabio Acquaviva, Fabrizio Barbetti, Elisabetta Caredda, Sergio Cocozza, Maurizio Delvecchio, Enza Mozzillo, Daniele Pirozzi, Francesco Prisco, Ivana Rabbone, Lucia Sacchetti, Nadia Tinto, Sonia Toni, Stefano Zucchini, Dario Iafusco, Italian Study Group on Diabetes of the Italian Society of Pediatric Endocrinology and Diabetology (ISPED)

**Affiliations:** 1 Department of Molecular Medicine and Medical Biotechnologies, University of Naples “Federico II”, Naples, Italy; 2 Laboratory of Molecular Endocrinology and Metabolism, Bambino Gesù Children's Hospital, Scientific Institute - Laboratory of Mendelian Diabetes, Rome, Italy; 3 Department of Pediatrics, Second University of Naples, Naples, Italy; 4 Pediatrics Unit, IRCCS Casa Sollievo della Sofferenza Hospital, S. Giovanni Rotondo (FG), Italy; 5 Department of Pediatrics, University of Naples “Federico II”, Naples, Italy; 6 CEINGE – Advanced Biotechnologies S. C. a R. L., Naples, Italy; 7 Department of Pediatrics, University of Turin, Turin, Italy; 8 Pediatric Diabetologic Unit, Meyer Children Hospital, Florence, Italy; 9 Department of Pediatrics, University of Bologna, Bologna, Italy; University of Texas Health Science Center at San Antonio, United States of America

## Abstract

MODY2 is the most prevalent monogenic form of diabetes in Italy with an estimated prevalence of about 0.5–1.5%. MODY2 is potentially indistinguishable from other forms of diabetes, however, its identification impacts on patients' quality of life and healthcare resources. Unfortunately, DNA direct sequencing as diagnostic test is not readily accessible and expensive. In addition current guidelines, aiming to establish when the test should be performed, proved a poor detection rate. Aim of this study is to propose a reliable and easy-to-use tool to identify candidate patients for MODY2 genetic testing. We designed and validated a diagnostic flowchart in the attempt to improve the detection rate and to increase the number of properly requested tests. The flowchart, called 7-iF, consists of 7 binary “yes or no” questions and its unequivocal output is an indication for whether testing or not. We tested the 7-iF to estimate its clinical utility in comparison to the clinical suspicion alone. The 7-iF, in a prospective 2-year study (921 diabetic children) showed a precision of about the 76%. Using retrospective data, the 7-iF showed a precision in identifying MODY2 patients of about 80% compared to the 40% of the clinical suspicion. On the other hand, despite a relatively high number of missing MODY2 patients, the 7-iF would not suggest the test for 90% of the non-MODY2 patients, demonstrating that a wide application of this method might 1) help less experienced clinicians in suspecting MODY2 patients and 2) reducing the number of unnecessary tests. With the 7-iF, a clinician can feel confident of identifying a potential case of MODY2 and suggest the molecular test without fear of wasting time and money. A Qaly-type analysis estimated an increase in the patients' quality of life and savings for the health care system of about 9 million euros per year.

## Introduction

Maturity-onset diabetes of the young type 2 (MODY2) is a monogenic form of diabetes with autosomic dominant transmission caused by heterozygous, inactivating mutation in the glucokinase gene (*GCK*). Loss-of-function *GCK* mutations impair glucose-sensing of the pancreatic beta cells (and liver) that in turn increase the threshold of pancreatic glucose-stimulated insulin secretion [1]–[3]. As a result, MODY2 patients have a moderate, not-progressive increase in fasting glucose and HbA1c levels and impaired glucose tolerance at the oral glucose tolerance test. In addition, carriers of *GCK* mutations are usually not prone to micro- or macro-vascular complications and, with rare exceptions, do not need pharmacological intervention [4].

It is now clearly established that MODY2 patients under treatment with insulin or oral anti-diabetic drugs can discontinue therapy without deterioration of their metabolic control. Moreover, inappropriate insulin treatment can induce iatrogenic weight gain and onset of insulin resistance (D.I., personal observation, and others [5], [6]). In fact, the typical MODY2 patient requires less frequent clinical surveillance than patients with other forms of diabetes. As a consequence, to diagnose MODY 2, especially in the pediatric age, is of paramount importance to ensure the appropriate clinical management and save healthcare resources [7].

The exact prevalence of MODY of any type is unknown, probably because their phenotypes overlap with the classical forms of diabetes. Their overall prevalence is estimated at around 1% of all diabetic cases, with MODY2 ranging from 30% to 60% of all MODY sub-types depending on clinical setting and geographic origin [8], [9]. Various studies indicate that only a small proportion of MODY are correctly identified. For instance, in the UK, only 5% to 20% of all patients theoretically affected by MODY are predicted to be correctly diagnosed [9], [10]. The number of patients referred for MODY genetic testing differs widely among diabetic clinics, and more experienced clinicians refer more patients for testing while keeping a good detection rate [9].

Genetic testing is highly specific and sensitive and represents the gold standard for diagnosing MODY2. However, this test has the drawback of being still expensive and not easily accessible. In 2008, the European Molecular Genetics Quality Network (EMGQN) published the guidelines for the selection of patients for MODY molecular testing, thereby providing an important common ground for European geneticists, pediatricians and diabetologists [5]. The guidelines included most of the relevant clinical parameters that define MODY, whereas others, such as the assay of the autoantibodies responsible for classical type 1 diabetes, were not taken into account [11], [12]. In this context, the guidelines have a poor detection rate and diagnostic power that can be improved, particularly in pediatrics, by adding other clinical criteria [13], [14].

Not all physicians treating diabetic patients have the expertise to recognize the clinical features of genetic forms of diabetes. Consequently, they hesitate to propose molecular testing, and it is likely that many patients remain undiagnosed. In this scenario, we designed and validated a 7-item flowchart (7-iF) to identify patients that have a high probability of carrying *GCK* mutations, thereby improving the detection rate and minimizing the number of unnecessary tests. We chose to study this specific type of MODY because is the commonest in Italy, at least in the pediatric population [15], [16], and because its diagnosis provides a perceptible impact on both patient's quality of life (no need of treatment) and health care costs (less frequent follow up visits, no stick for glycemic controls or drugs to provide). The 7-iF consists of seven questions with binary answers (yes/no) about data easily obtainable during a standard clinical examination, and therefore it can be applied in all clinical settings. The 7-iF includes the most recent criteria for the etiological diagnosis of diabetes (i.e. autoimmune diabetes antibodies, HbA1c levels and familiarity) and is particularly addressed to general practitioners and physicians working outside specialized centers, who are more prone to overlook this diagnosis. Here we show that the 7-iF has high clinical utility in comparison to the clinical suspicion alone and that a wide application of this method might 1) help less experienced clinicians in suspecting MODY2 patients and 2) reducing the number of unnecessary tests.

## Methods

### Ethics Approval

The study was conducted according to the Helsinki II declaration and it was approved by the Ethics Committee of the School of Medicine Federico II, Naples, Italy. Written informed consent to the study was obtained from each adult subject and from both parents of children.

### 7-item flowchart

We defined the seven clinical, biochemical and anamnestic criteria that best characterize a typical MODY2 patient: 1) negative test for pancreatic autoimmune markers; 2) insulin therapy naive; 3) HbA1c levels above or equal to 42 mmol/mol (HbA1c = 6%) on at least one occasion; 4) diabetes/hyperglycemia-onset ranging between 6 months and 25 years; 5) one parent affected by diabetes of any type (type 1, type 2 or gestational) or impaired fasting glucose (IFG) with or without impaired glucose tolerance (IGT); 6) no signs or symptoms suggestive of different types of diabetes (i.e., acanthosis nigricans, obesity, renal cysts, deafness and retinopathy); and 7) without concurring severe diseases, and not undergoing therapy that could impair glucose homeostasis (Table S1).

### Prospective evaluation of the 7-iF

We evaluated the clinical utility of the 7-iF in a “prospective cohort” consisting of 921 patients followed-up at the diabetic outpatient clinic of the Department of Pediatrics of the Second University of Naples (S.U.N.). The 7-iF was validated in two phases (Table 1).

**Table 1:** Validation of the 7-item flowchart.

	Step	N (%)
**PHASE I (Database query)**	Initial cohort	921 (100%)
	*Item 1*: Absence of autoimmune markers	310 (34%)
	*Item 2*: Absence of current or past insulin therapy	256 (28%)
	*Item 3*: HbA1c values ≥ 42 mmol/mol (6%)	54 (5.8%)
	Clinical re-evaluation	36 (3.9%)^a^
**PHASE II (Clinical re-evaluation)**	*Item 4*: Onset (diabetes or hyperglycemia) >6 m or <25 y	32 (3.5%)
	*Item 5*: Positive familiarity for either diabetes, IFG with or without IGT	30 (3.2%)
	*Item 6*: Absence of signs of other types of diabetes (acanthosis nigricans, deafness, renal cystis)	26 (2.8%)
	*Item 7*: Absence of other severe concurrent diseases	22 (2.4%)
	No relatives enrolled in the study	21 (2.3%)
	Molecular test	17 (1.8%)^b^
**Results**	Positive for *GCK* mutation	13 (1.4%)

Phase I: The electronic records of the patients were queried (Items 1–3). Phase II, each selected patient was clinically re-evaluated (Items 4–7). IFG, impaired fasting glucose; IGT, impaired glucose tolerance. ^a^First drop out; 18 patients were unreachable or refused to undergo clinical re-evaluation. ^b^Second drop out; 4 patients refused the genetic test.

### Phase 1: database query

The electronic database containing the patients' medical records was queried to answer the first three items of the 7-iF (autoimmunity, insulin therapy and HbA1c). All patients were tested for at least two of the following autoantibodies islet cell antibodies (ICA), glutamic acid decarboxylase (GAD), insulin auto-antibodies (IAA), the islet beta-cell-specific zinc transporter 8 (ZnT8) and the IA-2 protein. The cohort was further filtered to include only patients with no present or past insulin treatment and with HbA1c levels > 42 mmol/mol (6%) at least in one determination during their entire disease history. All patients who passed these filters were invited to proceed to phase 2.

### Phase 2: genetic and diabetologic counseling

We re-contacted selected patient and invited them to undergo further investigations. We performed a clinical re-evaluation, and genetic counseling of each compliant patient to obtain answers to the remaining 7-iF items (items 4–7). Patients fulfilling all the conditions were candidates for the genetic test. We prescribed the genetic test for only one individual per family. In the prospective cohort, we evaluated the precision of the 7-iF.

### Molecular test

Genomic DNA from the selected patients was extracted from a blood sample plus EDTA using the Nucleon BACC 2 kit (Amersham Biosciences Europe, Milan, Italy). Exons and flanking intron regions of GCK were amplified and sequenced as reported elsewhere [16] with GenBank NM_000162 as reference sequence. Variations were interpreted by comparison with those found in 100 non-diabetic Caucasian individuals of the same geographical origin and with known pathological variations identified by literature mining (www.hgmd.cf.ac.uk).

### Retrospective validation

**Figure 1:**
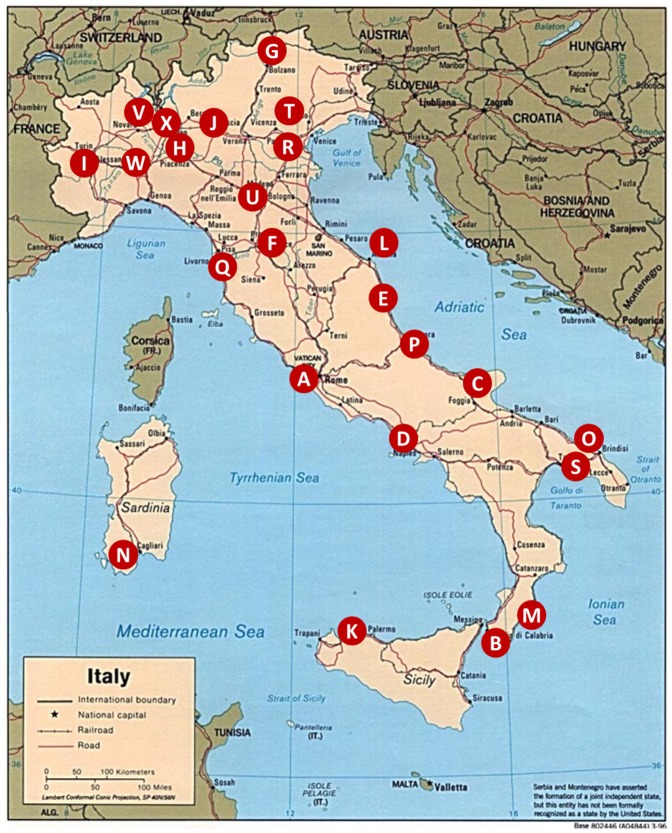
P.I. and city of each participating ISPED centers. A. Fabrizio Barbetti (Roma), B. Corrado Mammì (Reggio Calabria), C. Maurizio Delvecchio (San Giovanni Rotondo), D_1_. Nadia Tinto (Napoli), D_2_. Enza Mozzillo (Napoli), E. Luigi Pianese (Ascoli Piceno), F. Sonia Toni (Firenze), G. Bruno Pasquino (Bolzano), H. Valeria Calcaterra (Pavia), I. Ivana Rabbone (Torino), J. Barbara Felappi (Brescia), K. Francesca Cardella (Palermo), L. Valentino Cherubini (Ancona), M. Franco Mammì (Locri), N. Anna Paola Frongia (Cagliari), O. Francesco Gallo (Brindisi), P. Stefano Tumini (Chieti), Q. Sonia Lucchesi (Livorno), R. Carla Maria Monciotti (Padova), S. Susanna Coccioli (Francavilla Fontana), T. Amedeo Vergerio († deceased) (Feltre), U. Stefano Zucchini (Bologna), V. Francesco Cadario (Novara), Riccardo Lera (Alessandria), X. Andrea Scaramuzza (Milano).

We validated the clinical utility of the 7-iF on 372 patients (“first retrospective cohort”) addressed to the genetic test on the basis of clinical suspicion and with a MODY2 diagnosis confirmed by molecular test. This part of the study was conducted by physicians of the Italian Study Group on Diabetes of the Italian Society of Pediatric Endocrinology and Diabetology (ISPED) (Figure 1). The “first retrospective cohort” consisted of MODY2 patients from all over Italy to reduce population stratification and selection bias. The cohort consisted of patients for whom complete medical records were available; a single “unknown” to any of the 7-iF questions was considered an exclusion criterion. The “second retrospective cohort”, including 210 patients, was composed by the patients referred to (1) the Department of Molecular Medicine and Medical Biotechnologies, University of Naples “Federico II”- CEINGE and (2) the Pediatrics Unit, IRCCS Casa Sollievo della Sofferenza Hospital, S. Giovanni Rotondo (FG), with a clinical suspicion of MODY2 and the indication to the genetic test. We collected these latter data for patients referred in five years' time, before the beginning of the present study.

## Results

### Prospective evaluation of the 7-iF

To assess the clinical utility of the 7-iF, we evaluated clinical records of the 921 patients followed in the Diabetic Outpatient Clinic of the Department of Pediatrics of the Second University of Naples with biochemical data of the autoimmune markers for type 1 diabetes (GAD, ICA, IAA, IA2 and the ZnT8). As described in “Materials and Methods”, we applied a recursive filtering approach to select patients (Table 1). Patients positive for an item were queried about the next item. Among the 921 patients enrolled in the study, 21 (2.3%) fulfilled the 7 items and 13 (1.4%) had a positive genetic test. In detail, 310 (34%) patients were negative for the autoimmune markers and, 256/310 (83%) had not received insulin therapy. Of these 256 patients, 54 (21%) had HbA1c levels higher than or equal to 42 mmol/mol (HbA1c≥6%) on at least one occasion. Among these 54 patients invited to continue the study, 18 declined (unreachable or refused clinical re-evaluation). During the clinical re-evaluation, we excluded a further 15 patients: 4 because of a diabetes onset outside the age range; 2 without an affected parent; 4 for signs of different types of diabetes (1 with renal cysts, suspect MODY5; 3 with obesity and acanthosis nigricans, suspect early onset type 2 diabetes), 4 for concurrent severe diseases (2 with neurodevelopmental disorders and dysmorphic features, 1 with congenital dyserythropoietic anemia, 1 with acute lymphoblastic leukemia in remission) and 1 because a sibling in the study had already been identified as a candidate for the genetic test. Consequently, 21/921 (2.3%) patients were eligible for the test. Of these, 4 refused (unreachable or refused genetic test) and 17 underwent the test. The diagnosis was confirmed in 13/17 patients (Table 1), which corresponds to a precision of our test of about the 76%.

### Clinical features of the “prospective cohort”

The principal clinical features of all “prospective cohort” cases, both mutation negative and positive, are summarized in Tables S2 and S3. In a few cases, maximum HbA1c levels exceeded 53 mmol/mol (HbA1c = 7%); only in one case it exceeded 64 mmol/mol (HbA1c = 8%). Similarly, HbA1c level rarely fell below 42 mmol/mol (HbA1c = 6%), which confirms the slightly increased threshold of pancreatic glucose-stimulated insulin secretion. As expected, compared with the initial sample, MODY2 patients were younger at diagnosis (p<0.001) and had significantly lower values of maximum HbA1c (p = 0.019). MODY2 patients were generally lean, with a BMI z-score close to zero and maximum values usually below +1. On one occasion, a patient had increased body weight, with a BMI z-score within the obesity range (2.28), whereas his BMI z-score was usually around +1.

### Retrospective validation of the 7-iF

**Figure 2:**
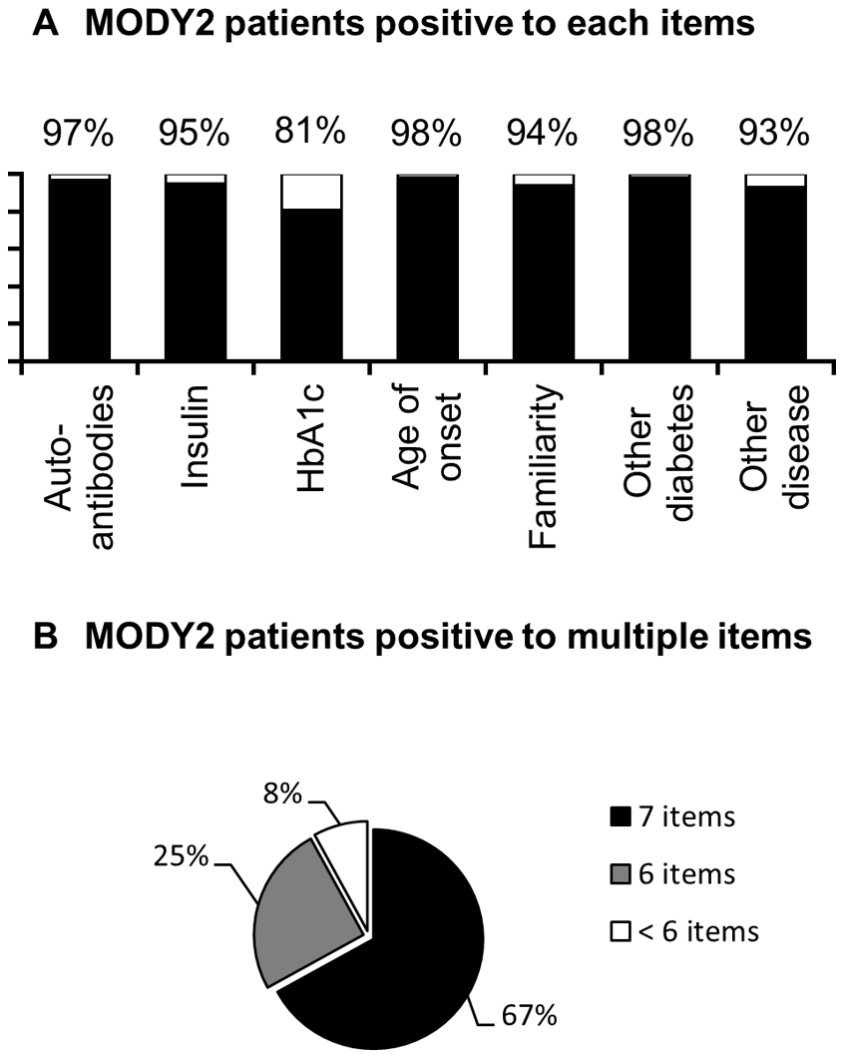
Validation on the “first retrospective cohort”. (A) Percentage of MODY2 patients positive to each item of the 7-iF. (B) Percentage of MODY2 patients positive to 7, 6 or less than 6 items of the 7-iF.

We tested and validated the 7-iF on a large series of MODY2 cases, the “first retrospective cohort”, recruited, merely on the basis of the clinical suspicion, by the members of the Study Group on Diabetes of the ISPED that is constituted by Italian hospital and university clinics caring children with diabetes. All these patients received a molecular diagnosis of MODY2 before the beginning of the present study. Data from 19 Italian outpatients' clinics and 5 medical genetics laboratories were pooled and analyzed (Figure 1). Each patient was unequivocally identified by a unique code to avoid overlapping. We received the records of 372 patients. Figure 2 summarizes the results of this retrospective validation. The upper panel shows the percentage of patients meeting each item. Most patients fulfilled each criterion. Although satisfactory (around 80%), the single item with the lowest prediction value was HbA1c. The lower panel shows the percentage of MODY2 patients fulfilling the 7 items, 6 out of 7 items or less than 6 items. About 67% of the MODY2 patients studied fulfilled 7 criteria out of 7, 25% of them proved positive to 6 out of 7 criteria and only 8% were positive just to 5 or 4 item of the 7-iF. This result indicates that, if strictly applied on this cohort of patients, the 7-iF would have correctly addressed to the genetic test the 67% of these patients missing 33% of them.

In order to validate the precision of the 7-iF, we investigate a “second retrospective cohort” composed by 210 pediatric diabetic patients referred, on the basis of the clinical suspicion, to two genetic laboratories, the Department of Molecular Medicine and Medical Biotechnologies, University of Naples “Federico II”- CEINGE and the Pediatrics Unit, IRCCS Casa Sollievo della Sofferenza Hospital, S. Giovanni Rotondo (FG), to perform the genetic test. All these patients were referred before the beginning of this study. Among this 210 patients, 85 (40%) received a confirmatory diagnosis of MODY2 whether 125 (60%) were MODY2 negative proving a precision of the clinical suspicion of 40% (85/210) with 60% (125/210) of patients erroneously addressed to the genetic test (false positive at the clinical suspicion). To evaluate the clinical utility of the 7-iF in comparison to the selection made upon the clinical suspicion, we calculated the 7-iF score for all the patients of this “second retrospective cohort”. Sixty-four patients (64/210; 30%) were positive to the 7-iF (score: 7/7) and among them 52 were MODY2 proving a precision of the 7-iF in identifying MODY2 patients of about 81% (52/64) similar to the 76% of the prospective evaluation. Among the 7-iF negative patients, 77% (113/146) resulted non MODY2 and 22% (33/146) MODY2. These 33 patients, corresponding to the about 39% of all the MODY2 patients, would have been missed by the 7-iF. On the other hand, 113 patients out of the 125 patients addressed to the genetic test and resulted negative were correctly suspected by the 7-iF as non-MODY2 (about 90% of true negative) further confirming the clinical utility of the 7-iF in helping less experienced clinicians in suspecting MODY2 patients.

## Discussion

The major issue with the MODY2 diagnosis is that, in general, physicians rarely suspect MODY2 and, also whether happen, many of them do not feel confident to ask for a molecular diagnosis. Some of the reasons can be the lacking of clear indications for the genetic test in the actual recommendations and the little experience that many physician have with rare monogenic disease. That might result in the estimated 80% of MODY2 patients without a correct diagnosed [9].

Here we propose a simple flowchart, the 7-iF, to identify candidates for the MODY2 genetic test. This flow-chart was conceived to be used by all physicians dealing with diabetes: it does not require specialized expertise in monogenic diabetes and is designed to be easily implemented in daily clinical practice in an outpatient setting. To explore its clinical utility, we evaluated its precision in prospective cohort and validated the results in two different retrospective cohorts. The 7-iF is broadly based on the EMGQN guidelines [5] but, unlike the latter, it includes T1D autoimmune markers and insulin treatment in the attempt to exclude patients with type 1 diabetes, which is the most prevalent form of diabetes (> 80%) in young individuals [11]. Consequently, a rationale search for different forms of diabetes is justified only when type 1 diabetes has been excluded. Although this strategy is not included in the EMGQN guidelines [5], many researchers and clinicians apply it [13], [14], [17], [18]. Regarding insulin treatment, most MODY2 patients have a modest impairment of glucose metabolism and usually do not need pharmacological intervention [17]. Therefore, patients with a positive history for insulin therapy are less likely to carry a pathological *GCK* mutation; consequently, only insulin-therapy naive patients are considered candidates for the genetic test.

The other additional criteria of the 7-iF with respect to the EMGQN guidelines are HbA1c levels, familiarity and age of onset. We used HbA1c instead of fasting glucose or the oral glucose tolerance test to select patients, because HbA1c reliably distinguishes between episodic hyperglycemia and persistent plasma glucose increase in the pathologic range. In fact, previous data [17], [19] and our clinical experience (Figure S1) indicate that a considerable proportion of MODY2 patients show a normal 2-hour glucose level at oral glucose tolerance tests. Indeed, recent guidelines for diabetes diagnosis include HbA1c as diagnostic marker [11], [20], although a consensus on the threshold value has yet to be reached. In the 7-iF, we use an HbA1c cut-off of 42 mmol/mol (HbA1c = 6%), which corresponds to a *sub-diabetes* state [20]. Based on the 7-iF, only patients with at least one parent affected by any alteration of glucose metabolism (type 1 or type 2 diabetes; gestational diabetes; IFG with or without IGT) are considered candidates for genetic testing. This criterion was included because MODY2 is an autosomal dominant inherited disease, with rare de novo mutations described [17]. Familiarity is not mandatory in the EMGQN guidelines [5], however recent studies found that it is the most powerful factor in distinguishing between MODY and sporadic forms of diabetes [10], [13], [14]. Furthermore, because MODY2 is usually discovered during infancy or in early adulthood, a diagnosis before 25 years of age is a criterion for the test in the 7-iF. Although a diagnosis of diabetes before 6 months of life is highly suggestive of a genetic cause, in such cases other types of monogenic diabetes (due to mutations in *KCNJ11, INS* or *ABCC8* genes) are more plausible and should be evaluated before MODY2 [21]. Finally, the 7-iF excludes from the genetic test, patients with complex, severe clinical conditions, such as syndromic diseases, cancers, organ failures. Although a *GCK* mutation can co-exist with other diseases, patients with such features should be first evaluated by specialists in genetic diabetes to determine whether their clinical conditions cause the altered glucose metabolism or a pancreatic deficiency really occurs.

In the present study, we showed that the precision of the 7-iF was sufficiently high (76%) to make the tool appropriate for routine application, especially if compared to precision of the ‘clinical suspect’ (40% estimated from the second retrospective cohort). Furthermore, also considering the stringent 7-iF criteria we diagnosed a number of patients not smaller than that expect in a random diabetic population. In fact, we recommended the test to 17 patients of the prospective cohort and MODY2 was diagnosed in 13 of them. This corresponds to a MODY2 frequency of 1.4% (13/921), which is consistent with the expected frequency (0.56%–1.56%) [9], [15].

Despite the relatively high number of potentially missed cases with the application of the 7-if in place of the clinical suspicion (about 33% and 39% from the first and the second retrospective cohort, respectively), considering the ability of the 7-iF in correctly identify 76% of MODY2 patients in a general pediatric diabetic population, together with the excellent ability in correctly identify those patients with low chances of being MODY2 (about 90% of true negative), we believe that a widely application of the 7-iF to all the diabetic patients will result in an overall increase of the MODY2 diagnosis. That because, the availability of clear indications will increase the number of requested tests and will result in a decrease of negative results (for the higher precision of the 7-iF compared to the clinical suspicion). On the other hand, lower score of positive items to the 7-iF (6/7) should not be considered exclusion criteria. These latter patients would need further observation and eventually to be referred to center with experience in monogenic diabetes, especially if they present clinical features suggestive of genetic forms of diabetes, such as the familiarity consistent with a dominant disease, similar clinical features among familiars, and no necessity of insulin therapy in the proband and other familiars even after many years of disease.

Recently, Shield et al. [10] proposed and validated a mathematical model that estimates the probability that a diabetic patient has of being affected by any of the three most common forms of MODY, and reported the probability that each patient had of being a carrier of a pathological mutation. At variance, the 7-iF serves to identify patients potentially affected specifically by MODY2 (the most frequent MODY subtype reported in Italy [15], [16]) Interestingly, when we tested our retrospective cohorts for the latter proposed model [10] we obtained less satisfactory specificity (10%). This observation supports the use of the 7-iF as a useful tool to improve the diagnosis of MODY2. Particularly, providing as output a “yes or no” indication to the molecular test, it is a very friendly to use during a standard clinical examination by general practitioners.

During the prospective evaluation, several patients refused to undergo further specialized diabetes and genetic investigations. These patients, negative for autoimmunity markers, affected by mild diabetes, without on-going therapy and with a good glycaemia control, probably felt that further clinical evaluations were not necessary. Considering the clinical characteristic of the typical MODY2 patient, we believe this group to be enriched for MODY2.

Detailed analysis of 7-iF output revealed that each item included was frequent in MODY2 patients. In fact, almost all 7 items elicited a “yes” answer in more than 90% of patients. Interestingly, we found that most (> 90%) of the MODY2 patients met at least 6 of the 7-IF criteria and that the “missing” criterion was not always the same (Figure 2A). This result suggests that a positive response to any 6 of the 7 criteria of the 7-iF should be considered an indication for the genetic test, irrespectively of which item is missing. However, the precision of 6/7 items should be prospectively evaluated before being implemented in clinical practice.

The single item with the lowest prediction value was HbA1c level with sensitivity around 80%; this is probably due to the high threshold that we imposed. We selected a threshold that we believed would balance two opposite goals: to test all patients with a high suspicion of being MODY and to reduce the number of tests to those with highest probability of being positive. This threshold was chosen according to the International Expert Committee's definition of sub-diabetes status [20]. Of note, a relevant number (15%) of *GCK*-mutation carriers evaluated in the first retrospective cohort showed maximum HbA1c values slightly below 42 mmol/mol (i.e.<6%). while less than 1% had HbA1c levels above 64 mmol/mol (i.e.>8%) (Figure S2). Potentially, lowering the HbA1c threshold to 38 mmol/mol (HbA1c = 5.6%), or even using a range of HbA1c values from 38 mmol/mol to 64 mmol/mol (5.6%>HbA1c<8%), would improve the ability to correctly identify potential MODY2 patients to address to the *GCK* genetic test. However, these changes will need a prospective and retrospective validation in different cohorts and we expect a certain reduction of the specificity and of the positive prediction value.

We believe that the 7-iF is a useful tool for all physicians dealing with diabetic patients including those working outside the context of a specialized center. Although the 7-iF “yes/no”-approach forces a continuous clinical spectrum into artificial stringent threshold categories, it is probably the best way to make it standardized and replicable. The power of this method is that it does not require specific knowledge about this form of diabetes. In fact, it could induce non-specialist clinicians, who are less prone to consider MODY2 diabetes, to re-evaluate their strategy of diagnosis. An implicit caveat is that more complex and uncertain cases should be referred to specialized centers.

From a cost-effectiveness viewpoint, we believe that widespread application of the 7-iF would result in optimization of healthcare resources. By using QALY analysis (Material S1), we estimated that the application of the 7-iF could provide an increase in the quality of life and a saving for the health system of about 3000 in a 10 years period for every MODY2 diagnosed. Considering an expected prevalence of at least 1% of MODY2 diabetic patients among the Italian diabetic population [22] this would account for a 9 million euro saving per year. Therefore, the initial cost of the genetic test would be easily offset by the savings resulting from the avoidance of life-long insulin therapy and three-month follow-ups. The 7-iF criteria are highly specific for MODY2 and with no extra costs except the autoantibody evaluations for each patient, which are in any event recommended.

In conclusion, we provide the first simple set of binary items for the identification of MODY2 patients that has been retrospectively and prospectively validated. We demonstrate that the 7-iF is reliable and with high precision in identifying MODY2 patients. It can be easily implemented in all clinical settings to select patients to undergo the MODY2 genetic test. This strategy will probably increase the number of diagnoses of MODY2 with a consequent considerable impact on the patient's quality of life and on healthcare resources.

## Supporting Information

Figure S1 **Oral Glucose Tolerance Test (OGTT) in MODY2.** Most GCK-MODY2 patients show basal level of blood glucose above the normal range and high fasting glucose levels (IFG) (basal glycaemia above 100 mg/dl). Nevertheless, a considerable proportion of MODY2 patients show normal 2-hours glucose level at oral glucose tolerance tests, with glycemic values within the normal range or the impaired glucose tolerance (IGT).
(JPG)

Figure S2 **Maximum HbA1c levels in MODY2 ascertained patients.** About the 80% of MODY2 patients investigated in the retrospective cohort had, at least in one occasion, HbA1c levels above or equal to 6% and, therefore, met the flowchart's criterion. Intriguingly a significant percentage of them (15%) showed maximum HbA1c records between 5.6 and 6%.
(PDF)

Table S1 The 7-item flowchart.
(PDF)

Table S2 Clinical features of patients selected for the genetic test in the prospective study.
(PDF)

Table S3 Clinical features of the patients in the prospective study. Please note: Four patients are not included because positive to the 7-iF but negative to the genetic test.
(PDF)

Material S1 The QALY analysis
(PDF)
