# Supplementary figures and images for: Identification of Candidate Children for Maturity-Onset Diabetes of the Young Type 2 (MODY2) Gene Testing: A Seven-Item Clinical Flowchart (7-iF)
Source: PLoS One. 2013 Nov 11;8(11):e79933. doi: 10.1371/journal.pone.0079933 (PMC3823596; doi:10.1371/journal.pone.0079933)

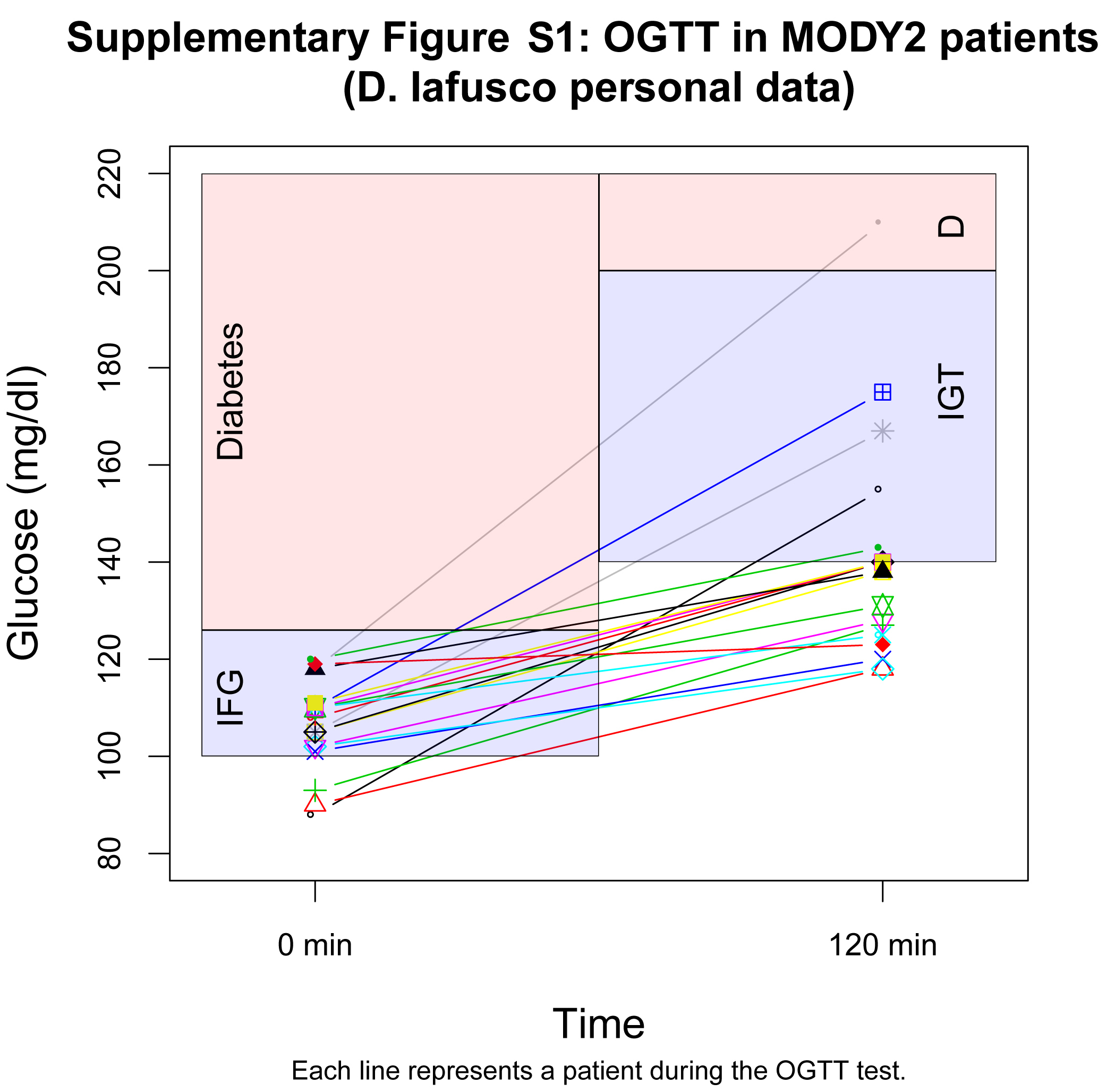

Supplement: Figure S1 — Oral Glucose Tolerance Test (OGTT) in MODY2. Most GCK-MODY2 patients show basal level of blood glucose above the normal range and high fasting glucose levels (IFG) (basal glycaemia above 100 mg/dl). Nevertheless, a considerable proportion of MODY2 patients show normal 2-hours glucose level at oral glucose tolerance tests, with glycemic values within the normal range or the impaired glucose tolerance (IGT). (JPG) [file pone.0079933.s001.jpg]

### Maximum HbA1c levels in MODY2 italian patients

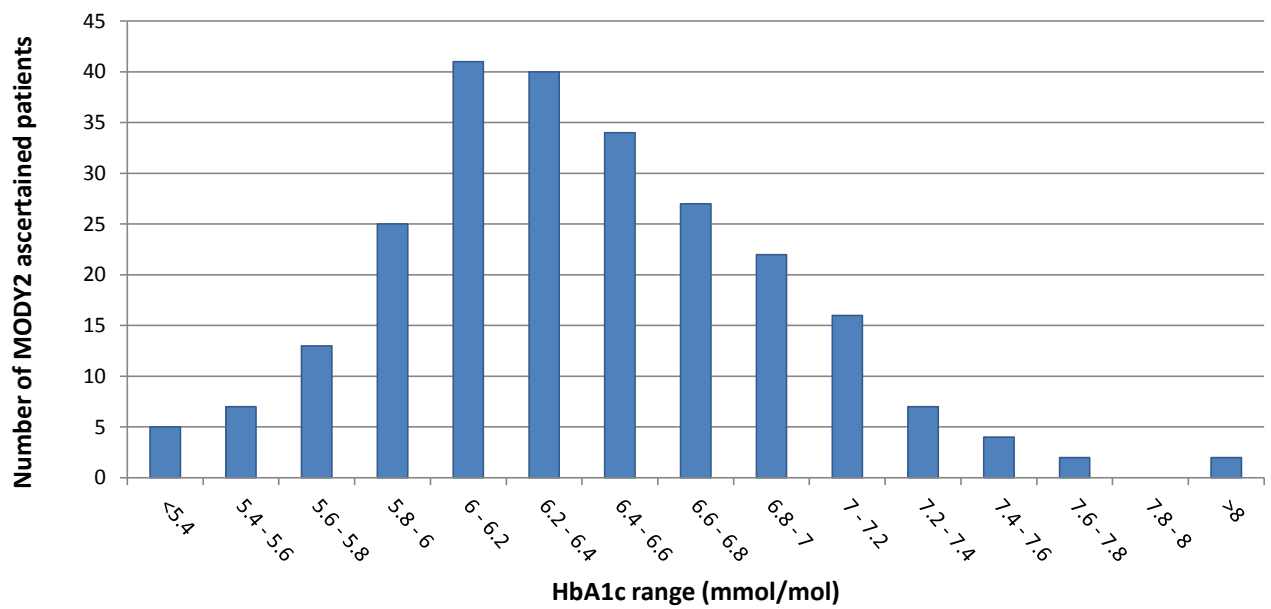

Supplement: Figure S2 — Maximum HbA1c levels in MODY2 ascertained patients. About the 80% of MODY2 patients investigated in the retrospective cohort had, at least in one occasion, HbA1c levels above or equal to 6% and, therefore, met the flowchart's criterion. Intriguingly a significant percentage of them (15%) showed maximum HbA1c records between 5.6 and 6%. (PDF) [file pone.0079933.s002.pdf]
