# Supplementary material for: Identification of Candidate Children for Maturity-Onset Diabetes of the Young Type 2 (MODY2) Gene Testing: A Seven-Item Clinical Flowchart (7-iF)
Source: PLoS One. 2013 Nov 11;8(11):e79933. doi: 10.1371/journal.pone.0079933 (PMC3823596; doi:10.1371/journal.pone.0079933)
Supplement: Table S1 — The 7-item flowchart. (PDF) [file pone.0079933.s003.pdf]

Supplementary table S1

7 ITEMS FLOWCHART (7IF)

Indications to GCK-MODY2 molecular tests

Disclaimer statement: Results obtained from the proposed questionnaire should never replace clinician's advice and opinion. The final decision about any diagnostic and therapeutic procedure is under the clinicians' responsibility.

1) Absence of autoimmune markers

Answer	Indication tor test
Negative marker	Yes
At least one marker positive	No
Markers not checked	No

2) Current or past insulin therapy

Answer	Indication tor test
No insulin therapy	Yes
Insulin therapy	No

3) HbA1c values

Answer	Indication tor test
HbA1c values ≥ 42 mMol/L (6%)	Yes
HbA1c values < 42 mMol/L (6%)	No

4) Onset (diabetes or hyperglycaemia) > 6 month or < 25 years

Answer	Indication tor test
After 6 months and before 25 years	Yes
Before 6 months of age	No
After 25 years of age	No

5) Positive familiarity for diabetes, IFG or IGT

Answer	Indication tor test
At least one parent affected	Yes
No affected parent	No

6) Signs of different type of diabetes

Answer	Indication tor test
None	Yes
Obesity (BMI > 30 Kg/m2 in adults or z-score > 2 in children)	No
Acanthosis nigricans	No
Renal cystis	No
Retinopathy or deafness	No

7) Other severe concurrent diseases

Answer	Indication tor test
No other disease	Yes
Other disease	No

Genetic test is indicated to patients with "Yes" to every item

Pinelli and Acquaviva et al. "Identification of candidate children for maturity-onset diabetes of the young type2 (MODY2) gene testing: A seven-item clinical flowchart (7-iF)"
