# Supplementary material for: Identification of Candidate Children for Maturity-Onset Diabetes of the Young Type 2 (MODY2) Gene Testing: A Seven-Item Clinical Flowchart (7-iF)
Source: PLoS One. 2013 Nov 11;8(11):e79933. doi: 10.1371/journal.pone.0079933 (PMC3823596; doi:10.1371/journal.pone.0079933)
Supplement: Table S2 — Clinical features of patients selected for the genetic test in the prospective study. (PDF) [file pone.0079933.s004.pdf]

Supplementary Table 2: Clinical features of patients selected for the genetic test in the prospective study.

***GCK* mutation**	**Gender**	**Age at onset (years)**	**HbA1c max (%)**	**HbA1c min (%)**	**BMI z-score max**	**BMI z-score min**
Positive	M	12	6.4	6.3	0.8	0.01
Positive	M	2.5	6.1	5.2	2.28	0.97
Positive	F	1.5	6.6	5.6	0.57	-0.14
Positive	F	6	6.9	6.3	0.49	-0.36
Positive	F	11.5	6.5	5.8	0.84	0.09
Positive	M	4	7.2	6.5	1.73	1.2
Positive	F	4	6.7	5.9	1.4	0.56
Positive	F	11.5	7.2	6.3	0.66	-0.38
Positive	F	4	7.2	6.3	0.17	-0.44
Positive	F	10	6.6	6.2	0.33	-0.95
Positive	M	5	8.2	6.0	0.47	0.22
Positive	F	6	6.7	6.7	0.85	0.85
Positive	M	8	7.2	6.4	0.12	-0.93
Negative	F	12	10.0	7.2	1.36	0.89
Negative	M	13	6.5	6.3	0.87	0.57
Negative	F	11	7.0	6.6	0.97	0.97
Negative	M	14	6.7	6.2	0.78	0.35
