# Supplementary material for: Identification of Candidate Children for Maturity-Onset Diabetes of the Young Type 2 (MODY2) Gene Testing: A Seven-Item Clinical Flowchart (7-iF)
Source: PLoS One. 2013 Nov 11;8(11):e79933. doi: 10.1371/journal.pone.0079933 (PMC3823596; doi:10.1371/journal.pone.0079933)
Supplement: Table S3 — Clinical features of the patients in the prospective study. Please note: Four patients are not included because positive to the 7-iF but negative to the genetic test. (PDF) [file pone.0079933.s005.pdf]

**Supplementary Table 3:** Clinical features of the patients in the prospective study

| | **7-iF negative patients** | **7-iF positive patients (GCK mutation positive)** | **Difference p-value[a]** |
|---|---|---|---|
| N | 904 | 13 | |
| Age in years ± SD | 14.3 ± 5.9 | 13.8 ± 5.0 | n.s. |
| Number of males (%) | 542 (60%) | 5 (38%) | n.s. |
| Age at discovery in years ± SD | 12.9 ± 5.9 | 6.6 ± 3.6 | <0.001 |
| Maximum BMI z-score ± SD | 0.67 ± 1.08 | 0.82 ± 0.69 | n.s. |
| Minimum BMI z-score ± SD | 0.00 ± 1.14 | 0.05 ± 0.75 | n.s. |
| Maximum HbA1c ± SD | 8.51 ± 2.48 | 6.9± 0.5 | 0.019 |
| Minimum HbA1c ± SD | 6.48 ± 1.23 | 6.1± 0.4 | n.s. |

Please note: Four patients are not included because positive to the 7-iF but negative to the genetic test. [a]n.s, not significant
