# Supplementary material for: Identification of Candidate Children for Maturity-Onset Diabetes of the Young Type 2 (MODY2) Gene Testing: A Seven-Item Clinical Flowchart (7-iF)
Source: PLoS One. 2013 Nov 11;8(11):e79933. doi: 10.1371/journal.pone.0079933 (PMC3823596; doi:10.1371/journal.pone.0079933)
Supplement: Material S1 — The QALY analysis (PDF) [file pone.0079933.s006.pdf]

QALY analysis

Condition 1:
MODY2 patients without a MODY2 diagnosis and wrongly tagged as either "to be determined hyperglycemia or as "diabetes"

Condition 2:
MODY2 patients with a genetic diagnosis of MODY2

Equation:
QALY = t * Q

t: time

Patients in both conditions are not different for lifespan expectancy, that should be considered the same of unselected population. However, we decided to limit the time to **10 years** because in a longer period other factors, related to either the patient itself or to the health system, can modify the quality of life. For instance, patients subjected to insulin injection can receive in the future less invasive treatments thanks to the advancement of medical technologies.

Q: utility value

Patients in condition 1
Patients in condition 1 are subjected to periodical clinical evaluations and blood analyses. All of them check daily, on capillary blood, the glicemic status and a small portion of them can even be wrongly treated with insulin. According to our data on the retrospective cohort, 5% of MODY2 patients have, indeed, been treated with insulin at least once in life, thus reducing their quality of life. A further reduction in their life-quality can derive by the emotional distress due to the knowledge of being affected by a chronic and progressive disease with potential severe consequence.
Hereby, we refer to untreated MODY2 patients as U and treated ones as T and to their utility values as QU and QT, respectively. We attribuited as utility value for untreated patients (QU) a value of 0.95 and for treated patients (QT) a value of 0.86 (adapted from: **Coffey et al, Diabetes Care 2002**). The resulting "Q" for patients in the condition 1 is: **Q1 = QU * 0.95 + QT * 0.05** (normalized to relative frequencies of QU and QT patients. In this formula 0.95 and 0.05 correspond, according to our data on the italian MODY2 population, to the 95% of MODY2 patients with no hystory of insulin therapy end to the 5% of them with hystory of insulin treatment).

QU	QT	Q1
0.95	0.86	0.9455

Patients in condition 2
Patients in the condition 2 usually undergo to a single peripheral blood sampling per year. Their quality of life is comparable to a non-diabetic subjects, because they should not receive any pharmacological intervention and should stop, or try to stop, insulin therapy. Furthermore, they have a only a moderate medical surveillance. Potential source of life-quality reduction could be the emotional distress related to the genetic test and the consciousness to have a disease potentially transmissible to their children. We, therefore, assessed their Q (Q2) as much close to the maximum possible.

Q2
0.99

The first result of the QALY analysis is the increase in quality of life for patients that receive a conclusive genetic diagnosis.

Q2-Q1
0.0445

Gain in quality of life **per year** per patient:

(Q2-Q1)/10
0.00445

Costs (in Euro)

In order to analyze the economical impact of the 7-iF, we calculated the costs for the health system per patients with and without a genetic diagnosis of MODY2. We refer, and adapt where necessary, to cost estimation recently published in the "Barometer Report 2012 - Italian Barometer Diabetes Observatory"

Patient in condition 1 with insulin therapy, 10 years expense

ET	8000

Patient in condition 1 without insulin therapy, 10 years expense:

EU	4000

All patients in condition 1, 10 years expenses:

**E1**	**4200**

(**8000*0.05 + 4000*0.95** --> where 0.95 and 0.05 correspond to the 95% of MODY2 patients without insulin therapy end to the 5% of them with hystory of insulin treatment)

Patient in condition 2, 10 yearly expense:

**E2**	**1500**

Absolute difference in health system expenses in 10 years time between patients with MODY2 genetic diagnosis and patients without it: $\mathbf{E_1 – E_2}$

**E1-E2**	**2700**

Health system expenses per QALY in a 10 years' time:

Condition 1: $R_1 = E_1 / Q_1$

R1	4442

Condition 2: $R_2 = E_2 / Q_2$

R2	1515

Difference in health system expenses in 10 years between patients with and without MODY2 genetic diagnosis taking in account also the gain in quality of life: R1 - R2

**R1-R2**	**2927**
